# ﻿Systematics and floral micromorphology of core *Aster* and its related groups (Asteraceae, Astereae): Taxonomic significance and phylogenetic insights

**DOI:** 10.3897/phytokeys.257.153925

**Published:** 2025-06-09

**Authors:** Xinyu Chen, Tianmeng Qu, Xinyi Zheng, Yanru Zhang, Hui Chen, Bo Li, Liang Dou, Zhixi Fu

**Affiliations:** 1 Key Laboratory of Land Resources Evaluation and Monitoring in Southwest (Sichuan Normal University), Ministry of Education, Chengdu 610066, China Sichuan Normal University Chengdu China; 2 College of Life Science, Sichuan Normal University, Chengdu 610101, China Sichuan Ecological Environment Monitoring Station Chengdu China; 3 Sichuan Ecological Environment Monitoring Station, Chengdu 610091, China Sichuan University Chengdu China; 4 Sichuan University Museum, Sichuan University, Chengdu 610065, China Sichuan Normal University Chengdu China; 5 The College of Life Sciences, Sichuan University, Chengdu 610065, China Sichuan Ecological Environment Monitoring Station Chengdu China; 6 Sustainable Development Research Center of Resources and Environment of Western Sichuan, Sichuan Normal University, Chengdu 610101, China Sichuan University Chengdu China

**Keywords:** *Aster*, Astereae, floral micromorphology, systematics, taxonomy

## Abstract

Four floral microcharacters were examined within 40 species of core *Aster* and its related groups in the genus *Aster* L. (Asteraceae, Astereae). These microcharacters included corolla lobe, style base, endothecium cell wall thickening, and the relative length of the stigmatic line to sterile tip appendage of style branch. Phylogenetic analysis was performed using internal transcribed spacers (ITS), followed by ancestral traits reconstruction based on four microcharacters. The phylogenetic results supported the inclusion of *Gymnaster*, *Heteropappus*, *Heteroplexis*, *Kalimeris*, and *Rhynchospermum* within core *Aster*, as well as the possible segregation of groups B–D into multiple new genera within Astereae. Groups A (9 species) and C (4 species) displayed both equally and unequally lobed corollas. Group D (5 species) exclusively exhibited equally lobed corollas. Groups A (26 species), B (1 species), and D (5 species) included both types of swollen and unswollen style bases. Group C (3 species) displayed only unswollen style bases. Additionally, the endothecium cell wall thickenings and relative length of the stigmatic line to the sterile appendage were both highly variable among groups A–D. Given the complex variation of these floral microcharacters, the results of floral micromorphology and ancestral trait reconstruction indicated that the taxonomic significance of floral micromorphology within core *Aster* and its related groups is limited. However, specific micromorphological traits (e.g., unequally lobed corolla, radial thickening, and longer relative length) may be still useful for diagnosing particular species or groups. Therefore, floral micromorphology should be integrated with other types of evidence for more accurate infrageneric classification of *Aster*.

## ﻿Introduction

The genus *Aster* L. (Asteraceae, Astereae) is widely distributed in Eurasia and comprises about 152–180 species (Nesom 1994a, 1994b; Chen et al. 2011; Chen et al. 2024). China is recognized as a major center of diversity for *Aster*, with approx. 123 species (Chen et al. 2011). The genus is characterized by white or purple ray florets and typically compressed achenes with 2–3 ribs (Nesom 1994a, 1994b; Anderberg et al. 2007). *Aster* was treated in a broad sense for a long time, encompassing hundreds of morphologically similar but distantly related species across Eurasia, Southeastern Africa, and North America (Nesom 1994a). Subsequent revisions, especially by Nesom (1994a), proposed the exclusion of North American (New World) asters and narrowed *Aster* to Old World species. Brouillet et al. (2009) further excluded African species from *Aster*. This revised circumscription has since been widely accepted by Brouillet et al. (2009), Chen et al. (2011), Li et al. (2012), and Chen et al. (2024). Chen et al. (2024) currently divided *Aster* into four clades: core *Aster* (Clade A), Asterser.Albescentes (Clade B), Asterser.Hersileoides (Clade C), and Alpine *Aster* (Clade D), proposing the potential independence of clades B–D as new genera. The core *Aster* was recognized as the groups including the “Amellus group” and “Kalimeris group” (Nesom 2020g). Although North American and African asters have been segregated into separate genera (except *Asteralpinus* L.), the precise circumscription of *Aster* remains unsettled and has been interpreted variably across studies. This suggests that, with further evidence, the delimitation of *Aster* may be revised comprehensively by incorporating related genera or segregating certain groups as new genera.

Ling et al. (1985) recognized that 12 genera in Astereae, e.g., *Gymnaster* Kitamm (*Miyamayomena* Kitamm), *Heteropappus* Less., *Heteroplexis* Chang, *Kalimeris* Cass., *Rhynchospermum* Reinw., are closely related to *Aster*. They also emphasized that *Aster* exhibits limited morphological variation. Based on involucre (layers, length, and texture) and arrangement of capitula, Ling et al. (1985) divided *Aster* into three groups: Astersect.Aster, Astersect.Orthomeris, and Astersect.Alpigenia. Nesom (1994a, 1994b) and Nesom and Robinson (2007) supported Ling et al. (1985) in proposing that *Aster* and its relatives (e.g., the Galatella group and the Kalimeris group) should be elevated to subtribe rank (Asterinae). However, Chen et al. (2011) proposed that some of 12 related genera should be included in *Aster* according to the analysis of Ling et al. (1985). Based on the color, number of ray florets and the layers of pappus, Chen et al. (2011) classified *Aster* into seven groups: Astersect.Teretiachaenium, Astersect.Ageratoides, Astersect.Asteromoea, Astersect.Asteromoea, Astersect.Pseudocalimeris, Astersect.Aster and Astersect.Alpigenia. The introduction of molecular data has provided new evidence for the taxonomic study of *Aster*. For instance, *Gymnaster* (Ito et al. 1995; Chen et al. 2011; Li et al. 2012; Nesom 2020d, 2020g), *Heteropappus* (Ito et al. 1998; Li et al. 2012; Nesom 2020d, 2020g; Duan et al. 2022; Chen et al. 2024), *Heteroplexis* (Nesom 2020d; Duan et al. 2022; Chen et al. 2024), *Kalimeris* (Ito et al. 1995; Ito et al. 1998; Chen et al. 2011; Li et al. 2012; Nesom 2020d, 2020g; Chen et al. 2024), and *Rhynchospermum* (Bremer and Anderberg 1994; Chen et al. 2011; Nesom 2020d, 2020g), were proposed for inclusion them within *Aster*. Many phylogenetic studies also revealed that the currently defined *Aster* may not be monophyletic (Selliah and Brouillet 2008; Pelser et al. 2010; Li et al. 2012; Jafari et al. 2015; Korolyuk et al. 2015; Fu et al. 2019). Li et al. (2012) proposed elevating Astersect.Alpigenia, Asterser.Albescentes, and Asterser.Hersileoides to generic rank based on ITS, ETS, and *trnL-F* markers. Similarly, Korolyuk et al. (2015) identified three monophyletic groups within *Aster*, including the Astherotamnus group, the typical Eurasian asters group, and the Heteropappus group. Currently, the taxonomic circumscription of *Aster* lacks sufficient support, with the existing phylogenetic relationships relying primarily on molecular evidence alone.

Compared with macromorphology, palynology, and cytology, floral micromorphology offers potential advantages including simple experimental procedures and additional sources of variation. King and Robinson (1970) elaborated “The New Synantherology” on the floral micromorphology of Asteraceae. Janaćković et al. (2019) proposed that floral micromorphology (e.g., style base, anther appendages) and anatomy are not old-fashioned approaches in the systematics of Asteraceae. They also suggested that it could provide substantial taxonomic evidence for subtribal and generic delimitation. For example, within the tribe Senecioneae, Liu and Yang (2010) examined several floral microcharacters (shape of the anther apical appendage, anther size, etc.) in 35 species and one variety of the genus *Sinosenecio* B.Nord. The result revealed that *Sinosenecio*, as previously defined, was polyphyletic, necessitating a taxonomic revision at the generic level. Within the tribe Vernonieae, Angulo et al. (2018) observed various microcharacters of the genus *Stenocephalum* Sch.Bip. (e.g., caudate anthers, pubescence of corollas, and apical appendages). These traits proved valuable for differentiating related species at both the generic and infrageneric levels. Within the tribe Cynareae, Gavrilović and Janaćković (2022) studied the micromorphology of CentaureaglaberrimaTauschsubsp.divergens (Vis.) Hayek. They identified specific microcharacters (glabrous corolla, short stylar hairs, etc.) that might have taxonomic significance and contribute to more precise species identification. Within the tribe Astereae, Sundberg (1985) evaluated several floral microcharacters (e.g., style base and endothecium) and found that their distribution does not correspond to existing generic or infrageneric boundaries. He concluded that relying solely on these microcharacters for generic delimitation would disrupt natural relationships and complicate the classification of the tribe. Based on comparisons of epidermal cells on the adaxial surface of ligules, ovary wall structure of ligulate flower, and basic chromosome number, Li et al. (2008) proposed that the genus *Sheareria* S.Moore should be included in the tribe Astereae instead of Heliantheae. Gao et al. (2009) further supported the taxonomic placement of Li et al. (2008) through revisions based on floral micromorphology (e.g., stamens and achene glandular hair) and molecular datasets (*trnL-F*, ITS). Moreover, increasing evidence has demonstrated the practical value of floral micromorphology in addressing systematic issues within various tribes such as Arctotideae (Karis et al. 2009), Cichorieae (Kilian et al. 2009), and Eupatorieae (Robinson and King 1977), as well as subtribes like Carduinae (Robinson 2009; Susanna and Garcia-Jacas 2009), Echinopinae (Robinson 2009; Susanna and Garcia-Jacas 2009), and Xerantheminae (Gavrilović et al. 2019).

In contrast to the examples above, research on the floral micromorphology in *Aster* has not been widely reported to date (Zhang et al. 2015, 2019; Xiao et al. 2020). A more extensive use of floral micromorphology data could aid the taxonomic understanding of this genus. Therefore, the data of floral micromorphology from 40 species of core *Aster* and its related groups were collected herein. This sampling includes 18 species recognized in eight new genera segregated from *Aster* by Nesom (2020a, 2020b, 2020d, 2020e, 2020f, 2020g, 2020h, 2020i) (Table 1). The objectives of this study are to: (i) report four floral micromorphological characters of *Aster* (corolla lobe, style base, endothecium cell wall thickening, and the relative length of stigmatic line to infertile tip appendage of style branch); (ii) reconstruct ancestral traits based on four floral micromorphological characters and the topology of the phylogenetic tree inferred from internal transcribed spacers (ITS); (iii) examine and evaluate the systematic implications of floral micromorphological data in the phylogeny of *Aster*.

**Table 1.** List of voucher specimens with different species names and corresponding collection localities.

Taxa	Species name used in this study	Name from Chen et al. (2011)	Name from Ling et al. (1985)	Name from the analyses of Nesom		Voucher specimens	Collection localities
Group A	*Astersmithianus* Hand.-Mazz.	*Astersmithianus* Hand.-Mazz.	*Astersmithianus* Hand.-Mazz.	*Astersmithianus* Hand.-Mazz.	Nesom (2020g)	Z. X. Fu 142	Zhuokeji Town, Barkam City, Sichuan Province, China
	*Astervestitus* Franch.	*Astervestitus* Franch.	*Astervestitus* Franch.	*Astervestitus* Franch.	Nesom (2020g)	G. J. Zhang 161	Shangri-la City, Yunnan Province, China
	*Asteraltaicus* Willd.	*Asteraltaicus* Willd.	*Heteropappusaltaicus* (Willd.) Novopokr.	*Asteraltaicus* Willd.	Nesom (2020g)	Z. X. Fu 13-0838	Fragrant Hill, Beijing City, China
	*Astermangshanensis* Y.Ling	*Astermangshanensis* Y.Ling	*Astermangshanensis* Y.Ling	*Astermangshanensis* Y.Ling	Nesom (2020g)	H. H. Hu 298	Yizhang county, Hunan Province, China
	*Asteroreophilus* Franch.	*Asteroreophilus* Franch.	*Asteroreophilus* Franch.	*Asteroreophilus* Franch.	Nesom (2020g)	Z. X. Fu 1803	Shangri-La City, Yunnan Province, China
	*Asterageratoides* Turcz.	/	*Asterageratoides* Turcz.	*Asterageratoides* Turcz.	Nesom (2020g)	Z. X. Fu 2027	Wudu City, Gansu Province, China
	*Asterverticillatus* (Reinw.) Brouillet	*Asterverticillatus* (Reinw.) Brouillet	*Rhynchospermumverticillatus* Reinw.	*Asterverticillatus* (Reinw.) Brouillet	Nesom (2020g)	Z. X. Fu 1641	Bomê County, Tibet, China
	*Asterturbinatus* S.Moore	*Asterturbinatus* S.Moore	*Asterturbinatus* S.Moore	*Asterturbinatus* S.Moore	Nesom (2020g)	H. H. Hu 312	Hangzhou City, Zhejiang Province, China
	*Asterpiccolii* Hook.f.	*Asterpiccolii* J.D.Hooker	*Gymnasterpiccolii* (Hook.f.) Kitam.	*Asterpiccolii* Hook.f.	Nesom (2020g)	Z. X. Fu 154	Taibai County, Shannxi Province, China
	*Astersinianus* Hand.-Mazz.	*Astersinianus* Hand.-Mazz.	*Astersinianus* Hand.-Mazz.	/	/	H. H. Hu 310	Xiushui County, Jiangxi Province, China
	*Astergiraldii* Diels	*Astergiraldii* Diels	*Astergiraldii* Diels	/	/	G. J. Zhang 196	Huyi District, Xi’an City, Shaanxi Province, China
	*Asteralatipes* Hemsl.	*Asteralatipes* Hemsl.	*Asteralatipes* Hemsl.	*Asteralatipes* Hemsl.	Nesom (2020e)	G. J. Zhang 206	Wushan County, Chongqing City, China
	*Astermoupinensis* (Franch.) Hand.-Mazz.	*Astermoupinensis* (Franch.) Hand.-Mazz.	/	/	/	G. J. Zhang 214	Beibei District, Chongqing City, China
	*Asterfalcifolius* Hand.-Mazz.	*Asterfalcifolius* Hand.-Mazz.	*Asterfalcifolius* Hand.-Mazz.	*Chaochienchangiafalcifolia* (Hand.-Mazz) Nesom	Nesom (2020i)	H. N. Qin et al. 18467	Shanghai City, China
	*Astertaiwanensis* Kitam.	*Astertaiwanensis* Kitam.	*Astertaiwanensis* Kitam.	*Astertaiwanensis* Kitam.	Nesom (2020g)	T. C. Chen et al. 12198	Taiwan Province, China
	*Asterbaccharoides* (Benth.) Steetz	*Asterbaccharoides* (Benth.) Steetz	*Asterbaccharoides* (Benth.) Steetz	*Asterbaccharoides* (Benth.) Steetz	Nesom (2020g)	S. Y. Hu 6242	Hong Kong S.A.R., China
	*Asterbrachytrichus* Franch.	*Asterbrachytrichus* Franch.	*Asterbrachytrichus* Franch.	*Tibetiodesbrachytricha* (Franch.) Nesom	Nesom (2020h)	Z. X. Fu 1091	Muli County, Sichuan Province, China
	*Asterprocerus* Hemsley	*Asterprocerus* Hemsley	/	*Asterprocerus* Hemsley	Nesom (2020d)	Z. X. Fu 693	Lin’an District, Hangzhou City, Zhejiang Province, China
	*Asterdolichopodus* Y.Ling	*Asterdolichopodus* Y.Ling	*Asterdolichopodus* Y.Ling	*Asterdolichopodus* Y.Ling	Nesom (2020g)	Z. X. Fu 100	Heping County, Guangdong Province, China
	*Astertianmenshanensis* G.J.Zhang & T.G.Gao	/	/	*Astertianmenshanensis* G.J.Zhang & T.G.Gao	Nesom (2020d)	H. H. Hu 310	Zhangjiajie City, Hunan Province, China
	*Asterindicus* L.	*Asterindicus* L.	Kalimerisindicavar.stenophylla Kitam.	*Asterindicus* L.	Nesom (2020g)	Z. X. Fu 739	Lechang City, Guangdong Province, China
	*Heteroplexismicrocephala* Y.L.Chen*	*Heteroplexismicrocephala* Y.L.Chen	/	/	/	H. H. Hu 392	Longzhou County, Guangxi Province, China
	*Astertientschwanensis* Hand.-Mazz.	*Astertientschwanensis* Hand.-Mazz.	/	*Tibetiodestientschwanensis* (Hand.-Mazz.) Nesom	Nesom (2020h)	G. J. Zhang 162	Tianquan County, Sichuan Province, China
	*Astersphaerotus* Y.Ling	*Astersphaerotus* Y.Ling	*Astersphaerotus* Y.Ling	*Astersphaerotus* Y.Ling	Nesom (2020e)	Z. X. Fu 162	Taibai County, Shaanxi Province, China
	*Asterdolichophyllus* Y.Ling	*Asterdolichophyllus* Y.Ling	*Asterdolichophyllus* Y.Ling	*Cardiagyrisdolichophylla* (Y.Ling) Nesom	Nesom (2020a)	Z. X. Fu 2002	Ruoergai County, Sichuan Province, China
	*Asterhispidus* Thunb.	*Asterhispidus* Thunb.	*Heteropappushispidus* (Thunb.) Less.	*Asterhispidus* Thunb.	Nesom (2020g)	Z. X. Fu 3121	Wenchuan County, Sichuan Province, China
Group B	*Asterhersileoides* C.K.Schneid.	*Asterhersileoides* C.K.Schneid.	*Asterhersileoides* Schneid.	*Yonglingiahersileoides* (C.K.Schneid.) Nesom	Nesom (2020i)	G. J. Zhang 171	Wenchuan County, Sichuan Province, China
Group C	*Asterlavandulifolius* Hand.-Mazz.	*Asterlavandulifolius* Hand.-Mazz.	/	*Sinosiduslavandulifolius* (Hand.-Mazz.) Nesom	Nesom (2020f)	Z. X. Fu 1605	Bomê County, Tibet, China
	*Asteralbescens* (DC.) Wall. ex Koehne	*Asteralbescens* (DC.) Wall. ex Koehne	*Asteralbescens* (DC.) Hand.-Mazz.	*Sinosidusalbescens* (DC.) Nesom	Nesom (2020f)	Z. X. Fu 1476	Nang County, Tibet, China
	*Asterhypoleucus* Hand.-Mazz.	*Asterhypoleucus* Hand.-Mazz.	*Asterhypoleucus* Hand.-Mazz.	*Sinosidushypoleucus* (Hand.-Mazz.) Nesom	Nesom (2020f)	G. J. Zhang 146	Nang County, Tibet, China
	*Asterargyropholis* Hand.-Mazz.	*Asterargyropholis* Hand.-Mazz.	*Asterargyropholis* Hand.-Mazz.	*Sinosidusargyropholis* (Hand.-Mazz.) Nesom	Nesom (2020f)	G. J. Zhang 153	Barkam City, Sichuan Province, China
Group D	*Asterbatangensis* Bureau & Franch.	*Asterbatangensis* Bureau & Franch.	*Asterbatangensis* Bur. et Franch.	*Geothamnusbatangensis* (Bureau & Franch.) Nesom	Nesom (2020f)	Z. X. Fu 7123	Muli County, Sichuan Province, China
	*Asterjeffreyanus* Diels	*Asterjeffreyanus* Diels	*Asterjeffreyanus* Diels	*Tibetiodesjeffreyana* (Diels) Nesom	Nesom (2020h)	Z. X. Fu 1699	Gongshan County, Yunnan Province, China
	*Astersalwinensis* Onno	*Astersalwinensis* Onno	*Astersalwinensis* Onno	*Tibetiodessalwinensis* (Onno) Nesom	Nesom (2020h)	Z. X. Fu 82	Shangri-la City, Yunnan Province, China
	*Astersetchuenensis* Franch.	*Astersetchuenensis* Franch.	/	*Tibetiodessetchuenensis* (Franch.) Nesom	Nesom (2020h)	Z. X. Fu 63	Li County, Sichuan Province, China
	*Asterprainii* (J.R.Drummond) Y.L.Chen	*Asterprainii* (J.R.Drummond) Y.L.Chen	*Asterprainii* (Drumm.) Y.L.Chen	*Chlamyditesprainii* J.R.Drummond	Nesom (2020b)	Z. X. Fu 3738	Qamdo City, Tibet, China
	*Asterfuscescens* Bureau & Franch.	*Asterfuscescens* Bureau & Franch.	*Asterfuscescens* Burr. et Franch.	*Griersoniafuscescens* (Bureau & Franchet) Nesom	Nesom (2020f)	Z. X. Fu 3437	Fugong County, Yunnan Province, China
	*Asterfarreri* W.W.Smith & Jeffrey	*Asterfarreri* W.W.Smith & Jeffrey	*Asterfarreri* W.W.Sm. et J.F.Jeffr.	*Tibetiodesfarreri* (W.W.Smith & Jeffrey) Nesom	Nesom (2020h)	Z. X. Fu 1848	Shangri-la County, Yunnan Province, China
	*Asterflaccidus* Bunge	*Asterflaccidus* Bunge	*Asterflaccidus* Bge.	*Tibetiodesflaccida* (Bunge) Nesom	Nesom (2020h)	Z. X. Fu 1747	Zayü County, Tibet, China
	*Asterhimalaicus* C.B.Clarke	*Asterhimalaicus* C.B.Clarke	*Asterhimalaicus* C.B.Clarke	*Tibetiodeshimalaica* (C.B.Clarke) Nesom	Nesom (2020h)	H. H. Hu 172	Shangri-la County, Yunnan Province, China

Notes. “/”: name not be included. “*”: treated as a member of *Aster* herein, as Nesom (2020d), Duan et al. (2022), and Chen et al. (2024) consistently concluded the inclusion of *Heteroplexis* in *Aster*. All voucher specimens are deposited in the PE Herbarium.

## ﻿Materials and methods

### ﻿Sampling, scientific names, and taxonomic treatments

The scientific names of the 40 sampled species in this study follow the nomenclature adopted in the “Flora of China” (Chen et al. 2011). The molecular systematic framework acknowledged in this study was proposed by Chen et al. (2024). To facilitate comparison of the taxonomic treatments in the “Flora Reipublicae Popularis Sinicae” (Ling et al. 1985), “Flora of China” (Chen et al. 2011), and the studies of Guy L. Nesom, a more visual data of species name, the number of voucher specimens, and their collection localities were provided in Table 1. With the advancement of morphological and molecular systematic studies, a thorough and formal taxonomic revision of *Aster* will be required in future studies.

### ﻿Floral micromorphology

**Figure 1.**
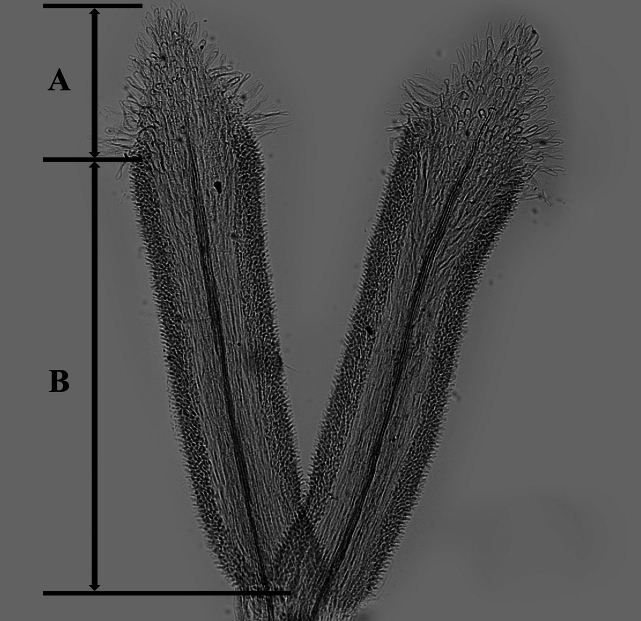
The morphology of style branch of the genus *Aster*, exemplified by *A.altaicus***A** the sterile tip appendage **B** the stigmatic line

**Figure 2.**
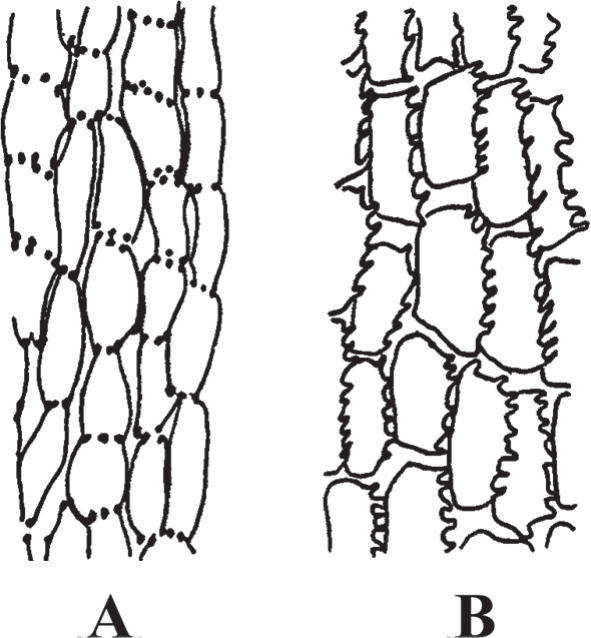
The types of cell wall thickenings in the tribe Senecioneae described by Robinson and Brettell (1973)**A** polarized **B** radial.

The descriptions of corolla lobes and style branches (Fig. 1) were adopted from Bremer and Anderberg (1994). The description of style base were adopted from Wetter (1983) and Sundberg (1985). The description of anther endothecial cell wall thickening (Fig. 2) was adopted from Dormer (1962), Nordenstam (1978), and Sundberg (1985). The mature capitula were taken from the herbarium specimens. The voucher specimens (Table 1) were all deposited in the Herbarium of Institute of Botany, Chinese Academy of Sciences (PE). The style tissue was dissected from mature disc florets to observe the swelling of style base. The tissue was then mounted on a slide and observed directly under a light microscope. For observing other microcharacters, the capitula were softened by boiling in distilled water for 5 minutes. The style, anther, and corolla of disc floret were sectioned using stereoscopy. They were transferred and fixed in FAA stationary liquid for 2 hours (anhydrous ethanol: acetic acid: formaldehyde = 8:1:1). The materials were rinsed several times with distilled water. They were then transferred to a 5% sodium hydroxide (NaOH) solution for 8 hours. Afterward, they were thoroughly rinsed with water. The materials were observed and dissected under a stereoscope and sealed with Hoyer solution. Finally, they were examined and photographed using an optical microscope. The data of four floral micromorphology generated were provided in Table 2.

**Table 2.** Floral micromorphology of the genus *Aster*.

Taxa	Species name	corolla lobe	reference figure	style base	reference figure	endothecium cell wall thickenings	reference figure	style branch	reference figure
Group A	*Astersmithianus*	/	/	swollen	Fig. 8A	polarized, radial	Fig. 13A	the length: stigmatic lines = Infertility tip appendage	Fig. 18A
	*Astervestitus*	/	/	unswollen	Fig. 8B	polarized	Fig. 13B	the length: stigmatic lines > Infertility tip appendage	Fig. 18B
	*Asteraltaicus*	/	/	unswollen	Fig. 8C	polarized, radial	Fig. 13C	the length: stigmatic lines > Infertility tip appendage	Fig. 18C
	*Astermangshanensis*	unequal	Fig. 4A	unswollen	Fig. 8D	polarized	Fig. 13D	the length: stigmatic lines = Infertility tip appendage	Fig. 18D
	*Asteroreophilus*	unequal	Fig. 4B	swollen	Fig. 8E	polarized, radial	Fig. 13E	the length: stigmatic lines < Infertility tip appendage	Fig. 18E
	*Asterageratoides*	unequal	Fig. 4C	unswollen	Fig. 8F	polarized, radial	Fig. 13F	the length: stigmatic lines < Infertility tip appendage	Fig. 18F
	*Asterverticillatus*	/	/	unswollen	Fig. 8G	polarized, radial	Fig. 13G	the length: stigmatic lines > Infertility tip appendage	Fig. 18G
	*Asterturbinatus*	unequal	Fig. 4D	swollen	Fig. 8H	polarized	Fig. 13H	the length: stigmatic lines = Infertility tip appendage	Fig. 18H
	*Asterpiccolii*	/	/	swollen	Fig. 8I	radial	Fig. 13I	the length: stigmatic lines > Infertility tip appendage	Fig. 18I
	*Astersinianus*	equal	Fig. 4E	unswollen	Fig. 8J	polarized, radial	Fig. 13J	the length: stigmatic lines > Infertility tip appendage	Fig. 18J
	*Astergiraldii*	equal	Fig. 4F	swollen	Fig. 8K	polarized, radial	Fig. 13K	the length: stigmatic lines = Infertility tip appendage	Fig. 18K
	*Asteralatipes*	unequal	Fig. 4G	swollen	Fig. 8L	polarized, radial	Fig. 13L	the length: stigmatic lines = Infertility tip appendage	Fig. 18L
	*Astermoupinensis*	unequal	Fig. 4H	swollen	Fig. 8M	polarized	Fig. 13M	the length: stigmatic lines = Infertility tip appendage	Fig. 18M
	*Asterfalcifolius*	/	/	swollen	Fig. 8N	polarized	Fig. 13N	the length: stigmatic lines = Infertility tip appendage	Fig. 18N
	*Astertaiwanensis*	/	/	swollen	Fig. 8O	radial	Fig. 13O	the length: stigmatic lines = Infertility tip appendage	Fig. 18O
	*Asterbaccharoides*	unequal	Fig. 4I	unswollen	Fig. 8P	polarized	Fig. 13P	the length: stigmatic lines > Infertility tip appendage	Fig. 18P
	*Asterbrachytrichus*	/	/	unswollen	Fig. 8Q	polarized, radial	Fig. 13Q	the length: stigmatic lines = Infertility tip appendage	Fig. 18Q
	*Asterprocerus*	/	/	unswollen	Fig. 8R	polarized, radial	Fig. 13R	the length: stigmatic lines > Infertility tip appendage	Fig. 18R
	*Asterdolichopodus*	/	/	unswollen	Fig. 8S	radial	Fig. 13S	the length: stigmatic lines < Infertility tip appendage	Fig. 18S
	*Astertianmenshanensis*	/	/	unswollen	Fig. 8T	polarized, radial	Fig. 13T	the length: stigmatic lines < Infertility tip appendage	Fig. 18T
	*Asterindicus*	/	/	swollen	Fig. 8U	polarized, radial	Fig. 13U	the length: stigmatic lines = Infertility tip appendage	Fig. 18U
	*Heteroplexismicrocephala*	/	/	unswollen	Fig. 8V	polarized, radial	Fig. 13V	the length: stigmatic lines > Infertility tip appendage	Fig. 18V
	*Astertientschwanensis*	/	/	swollen	Fig. 8W	polarized	Fig. 13W	the length: stigmatic lines = Infertility tip appendage	Fig. 18W
	*Astersphaerotus*	/	/	swollen	Fig. 8X	polarized	Fig. 13X	the length: stigmatic lines > Infertility tip appendage	Fig. 18X
	*Asterdolichophyllus*	/	/	unswollen	Fig. 8Y	polarized	Fig. 13Y	the length: stigmatic lines > Infertility tip appendage	Fig. 18Y
	*Asterhispidus*	/	/	swollen	Fig. 8Z	polarized, radial	Fig. 13Z	the length: stigmatic lines > Infertility tip appendage	Fig. 18Z
Group B	*Asterhersileoides*	/	/	swollen	Fig. 9	polarized	Fig. 14	the length: stigmatic lines > Infertility tip appendage	Fig. 19
Group C	*Asterlavandulifolius*	equal	Fig. 5A	unswollen	Fig. 10A	radial	Fig. 15A	the length: stigmatic lines > Infertility tip appendage	Fig. 20A
	*Asteralbescens*	unequal	Fig. 5B	unswollen	Fig. 10B	polarized, radial	Fig. 15B	the length: stigmatic lines = Infertility tip appendage	Fig. 20B
	*Asterhypoleucus*	equal	Fig. 5C	unswollen	Fig. 10C	polarized, radial	Fig. 15C	/	/
	*Asterargyropholis*	equal	Fig. 5D	/	/	polarized, radial	Fig. 15D	the length: stigmatic lines = Infertility tip appendage	Fig. 20C
Group D	*Asterbatangensis*	equal	Fig. 6A	swollen	Fig. 11A	polarized, radial	Fig. 16A	the length: stigmatic lines > Infertility tip appendage	Fig. 21A
	*Asterjeffreyanus*	/	/	swollen	Fig. 11B	polarized, radial	Fig. 16B	the length: stigmatic lines < Infertility tip appendage	Fig. 21B
Group D	*Astersalwinensis*	equal	Fig. 6B	unswollen	Fig. 11C	polarized	Fig. 16C	the length: stigmatic lines = Infertility tip appendage	Fig. 21C
	*Astersetchuenensis*	equal	Fig. 6C	unswollen	Fig. 11D	polarized, radial	Fig. 16D	the length: stigmatic lines = Infertility tip appendage	Fig. 21D
	*Asterprainii*	/	/	unswollen	Fig. 11E	polarized, radial	Fig. 16E	the length: stigmatic lines > Infertility tip appendage	Fig. 21E
	*Asterfuscescens*	/	/	/	/	polarized, radial	Fig. 16F	/	/
	*Asterfarreri*	/	/	/	/	polarized	Fig. 16G	/	/
	*Asterflaccidus*	equal	Fig. 6D	/	/	polarized, radial	Fig. 16H	the length: stigmatic lines = Infertility tip appendage	Fig. 21F
	*Asterhimalaicus*	equal	Fig. 6E	/	/	polarized	Fig. 16I	/	/

Note. “/”: Trait not be observed.

### ﻿Phylogenetic analysis

An ITS dataset including 30 *Aster* species and 2 outgroups (*Nannoglottiscarpesioides* Maxim. and *Nannoglottisgynura* (C.Winkl.) Y.Ling & Y.L.Cnen) was downloaded from NCBI GenBank database (Table 3). The data matrix was aligned by MAFFT v.7.526-Linux (Katoh et al. 2019) with the “--auto” strategy and subsequently trimmed for gaps by trimAl v.1.5.0-Linux (Capella-Gutierrez et al. 2009). Phylogenetic analyses were performed using Maximum Likelihood (ML) method in RAxML-NG v.1.2.2-Linux (Kozlov et al. 2019) with 1000 bootstrap replicates and Bayesian Inference (BI) in MrBayes v.3.2.7-Linux (Ronquist et al. 2012), both under the “SYM+G4” substitution model predicted by ModelTest-NG v.0.1.7-Linux (Darriba et al. 2020). In the BI analysis, 4 Markov chain Monte Carlo (MCMC) chains were run for 10,000,000 generations each, with sampling every 1,000 generations, starting with a random tree. After discarding the first 25% of samples, Bayesian posterior probabilities (PP) were calculated for the majority consensus tree based on all sampled trees. Finally, the phylogenetic tree was visualized and formatted in the program FigTree v.1.4.4 (http://tree.bio.ed.ac.uk/software/figtree).

**Table 3.** Taxa sampled and corresponding NCBI GenBank accession numbers of ITS sequences used in the phylogenetic analysis.

Taxa	Species name	NCBI GenBank accession number
Group A	*Astersmithianus*	MH710810.1
	*Astervestitus*	JN543769.1
	*Astermangshanensis*	JN543760.1
	*Asteroreophilus*	JN543826.1
	*Asterageratoides*	MH711534.1
	*Asterverticillatus*	JN543706.1
	*Asterturbinatus*	JN543814.1
	*Asterpiccolii*	MH710712.1
	*Astermoupinensis*	MH807113.1
	*Asterfalcifolius*	JN543802.1
	*Astertaiwanensis*	LC482291.1
	*Asterbaccharoides*	JN543805.1
	*Asterprocerus*	KP313683.1
	*Asterdolichopodus*	JN543775.1
	*Astertianmenshanensis*	KP313679.1
	*Asterindicus*	EF108396.1
	*Heteroplexismicrocephala*	MW703707.1
	*Asterdolichophyllus*	MH807107.1
	*Asterhispidus*	HQ154044.1
Group B	*Asterhersileoides*	JN543787.1
Group C	*Asterlavandulifolius*	JN543796.1
	*Asteralbescens*	MH808093.1
	*Asterargyropholis*	JN543793.1
Group D	*Asterbatangensis*	JN543859.1
	*Astersalwinensis*	ON527425.1
	*Astersetchuenensis*	JN543850.1
	*Asterfuscescens*	JN543751.1
	*Asterfarreri*	MH808095.1
	*Asterflaccidus*	JN543844.1
	*Asterhimalaicus*	PP746026.1
Outgroups	*Nannoglottiscarpesioides*	AY017161.1
	*Nannoglottisgynura*	AY017162.1

### ﻿Ancestral trait reconstruction

The character data used for ancestral traits reconstruction were based on four floral micromorphological traits observed from herbarium specimens collected by Associate Prof. Dr. Zhixi Fu and colleagues, held at PE. The character state codes associated with these data include (a) corolla lobe: (0) equal, (1) unequal; (b) style base: (0) swollen, (1) unswollen; (c) endothecium cell wall thickenings: (0) polarized, (1) radial, (2) both polarized and radial; (d) style branch: (1) the length: stigmatic lines = Infertility tip appendage, (2) the length: stigmatic lines > Infertility tip appendage, (3) the length: stigmatic lines < Infertility tip appendage. Two outgroups (*Nannoglottiscarpesioides* and *N.gynura*) were removed from ancestral state reconstruction due to the absence of observations of them for four floral micromorphological characters. The detailed code list for floral micromorphology used in ancestral trait reconstruction was also provided in Table 4. Based on the topology of the phylogenetic tree obtained, Mesquite v.3.8.1 (https://www.mesquiteproject.org) was applied to reconstruct ancestral states of core *Aster* and its related groups. The “Trace Character Over Trees” function and the “Likelihood Ancestral States” method, with the One-parameter Markov k-state probability (Mk1) model (Lewis 2001), was applied in Mesquite.

**Table 4.** Character state codes for floral micromorphology used in ancestral trait reconstruction.

Taxa	Species name	corolla lobe	style base	endothecium cell wall thickenings	style branch
Group A	*Astersmithianus*	/	0	2	0
	*Astervestitus*	/	1	0	1
	*Astermangshanensis*	1	1	0	0
	*Asteroreophilus*	1	0	2	2
	*Asterageratoides*	1	1	2	2
	*Asterverticillatus*	/	1	2	1
	*Asterturbinatus*	1	0	0	0
	*Asterpiccolii*	/	0	1	1
	*Astermoupinensis*	1	0	0	0
	*Asterfalcifolius*	/	0	0	0
	*Astertaiwanensis*	/	0	1	0
	*Asterbaccharoides*	1	1	0	1
	*Asterprocerus*	/	1	2	1
	*Asterdolichopodus*	/	1	1	2
	*Astertianmenshanensis*	/	1	2	2
	*Asterindicus*	/	0	2	0
	*Heteroplexismicrocephala*	/	1	2	1
	*Asterdolichophyllus*	/	1	0	1
	*Asterhispidus*	/	0	2	1
Group B	*Asterhersileoides*	/	0	0	1
Group C	*Asterlavandulifolius*	0	1	1	1
	*Asteralbescens*	1	1	2	0
	*Asterargyropholis*	0	/	2	0
Group D	*Asterbatangensis*	0	0	2	1
	*Astersalwinensis*	0	1	0	0
	*Astersetchuenensis*	0	1	2	0
	*Asterfuscescens*	/	/	2	/
	*Asterfarreri*	/	/	0	/
	*Asterflaccidus*	0	/	2	0
	*Asterhimalaicus*	0	/	0	/

## ﻿Results

### ﻿Molecular phylogenetic relationship

**Figure 3.**
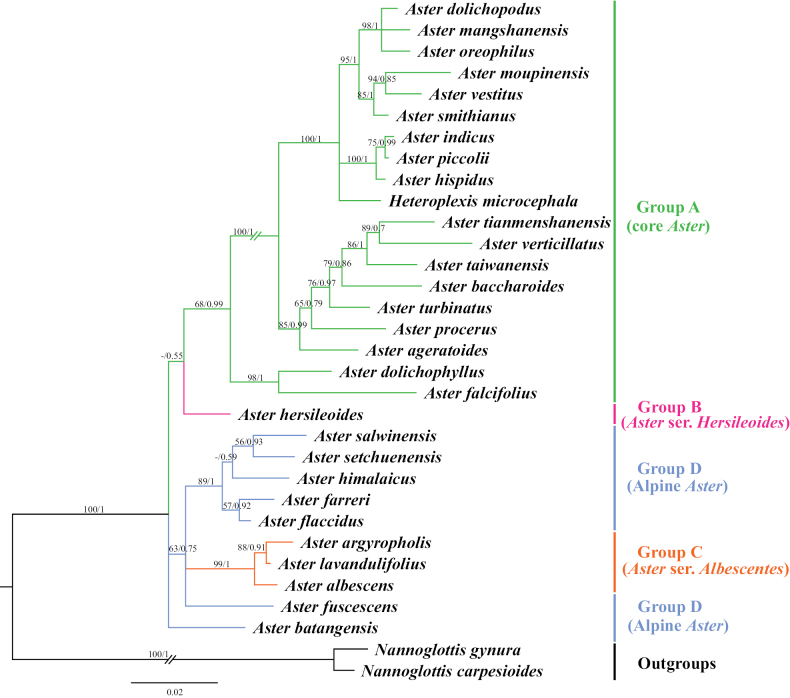
Molecular phylogenetic tree of 30 *Aster* and 2 outgroups species based on ITS sequences using Maximum Likelihood and Bayesian Inference methods. Species are color-coded into four groups according to Chen et al. (2024). Values above branches represent bootstrap (BS) and posterior probabilities (PP). “-” indicates that BS values are < 50%. Groups B–D may should be considered as several new genera.

The phylogenetic trees (Fig. 3) were constructed using ML and BI methods based on ITS sequences from 32 species, with bootstrap values (BS) and posterior probabilities (PP) indicated at the branches. The two resulting trees exhibited identical topologies. Most branches displayed strong support values. All sampled taxa represented four groups from *Aster*, including core *Aster* (group A), Asterser.Hersileoides (group B), Asterser.Albescentes (group C), and Alpine *Aster* (group D) (Fig. 3). The core *Aster* (19 species) formed a monophyletic group with strong support (PP = 0.99). The result also supported the inclusion of *Gymnaster* (*A.piccolii*), *Heteropappus* (*A.altaicus*, *A.hispidus*), *Heteroplexis* (*H.microcephala*), *Kalimeris* (*A.indicus*), and *Rhynchospermum* (*A.verticillatus*) within core *Aster*. Asterser.Hersileoides (*A.hersileoides*) formed a separate group with low support (PP = 0.55). Asterser.Albescentes (*A.albescens*, *A.argyropholis*, and *A.lavandulifolius*) and Alpine *Aster* (6 species, *A.batangensis* excluded) clustered together into a group with moderate support (BS/PP value = 63/0.75). The result indicated that Asterser.Albescentes is closely related to Alpine *Aster* group. Notably, *A.batangensis* formed a distinctive lineage with strong support (BS/PP = 100/1) and was not nested within Alpine *Aster*. It was positioned at the base of the sampled species, possibly representing a potential basal species within *Aster*. As a result, the weak support for the sister relationship between *A.hersileoides* and core *Aster* suggested that Asterser.Hersileoides, Asterser.Albescentes, and Alpine *Aster* are distantly related to core *Aster*.

### ﻿Corolla lobe

**Figure 4.**
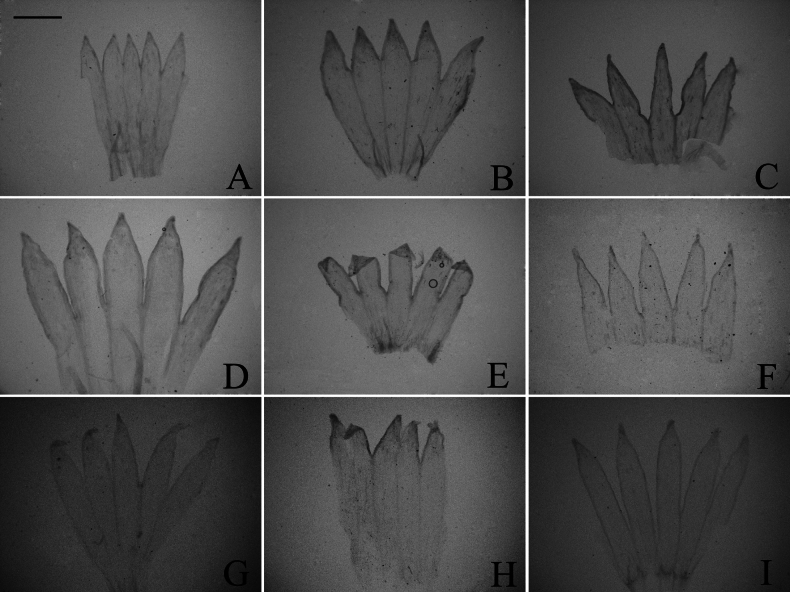
The morphology of corolla lobe in group A **A***A.mangshanensis***B***A.oreophilus***C***A.ageratoides***D***A.turbinatus***E***A.sinianus***F***A.giraldii***G***A.alatipes***H***A.moupinensis***I***A.baccharoides*. Scale bar: 1 mm.

**Figure 5.**
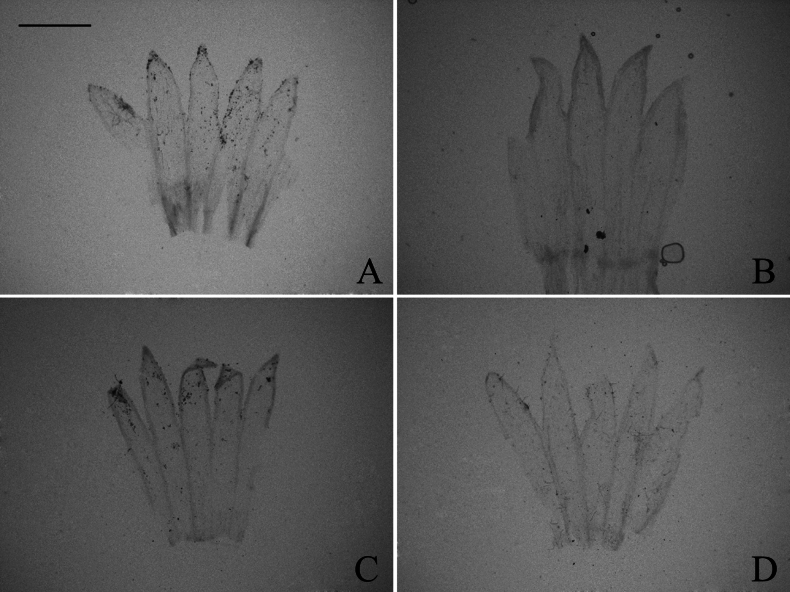
The morphology of corolla lobe in group C **A***A.lavandulifolius***B***A.albescens***C***A.hypoleucus***D***A.argyropholis*. Scale bar: 1 mm.

**Figure 6.**
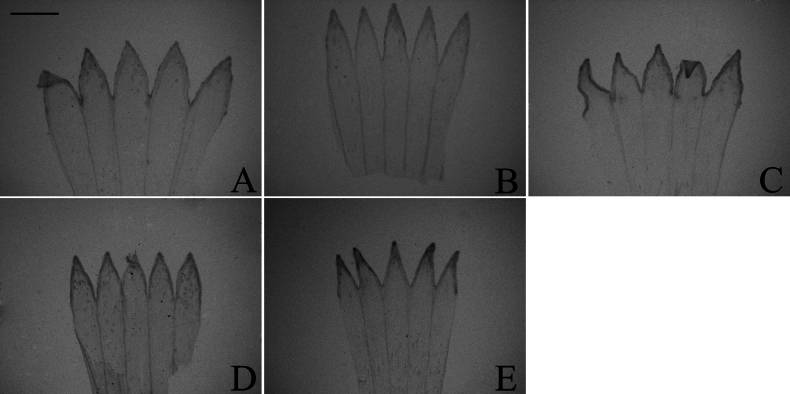
The morphology of corolla lobe in group D **A***A.batangensis***B***A.salwinensis***C***A.setchuenensis***D***A.flaccidus***E***A.himalaicus*. Scale bar: 1 mm.

The corolla of all taxa examined is five-lobed. It was categorized into two types: equally lobed and unequally lobed. In our study, both types of corollas were found in group A and group C (Figs 4, 5), while only equally lobed type was present in group D (Fig. 6). Considering the insufficient, evident systematic value of corolla lobe in the initial observations, further examination of the remained species was discontinued. Within group A, the corolla was equally lobed in *A.sinianus* (Fig. 4E) and *A.giraldii* (Fig. 4F). The corolla was unequally lobed in 7 species (Fig. 4A–D, G–I). Within group C, the corolla was equally lobed in *A.lavandulifolius* (Fig. 5A), *A.hypoleucus* (Fig. 5C), and *A.argyropholis* (Fig. 5D). The corolla was unequally lobed in *A.albescens* (Fig. 5B). Within group D, the corolla was equally lobed in *A.batangensis* (Fig. 6A), *A.salwinensis* (Fig. 6B), *A.setchuenensis* (Fig. 6C), *A.flaccidus* (Fig. 6D), and *A.himalaicus* (Fig. 6E).

**Figure 7.**
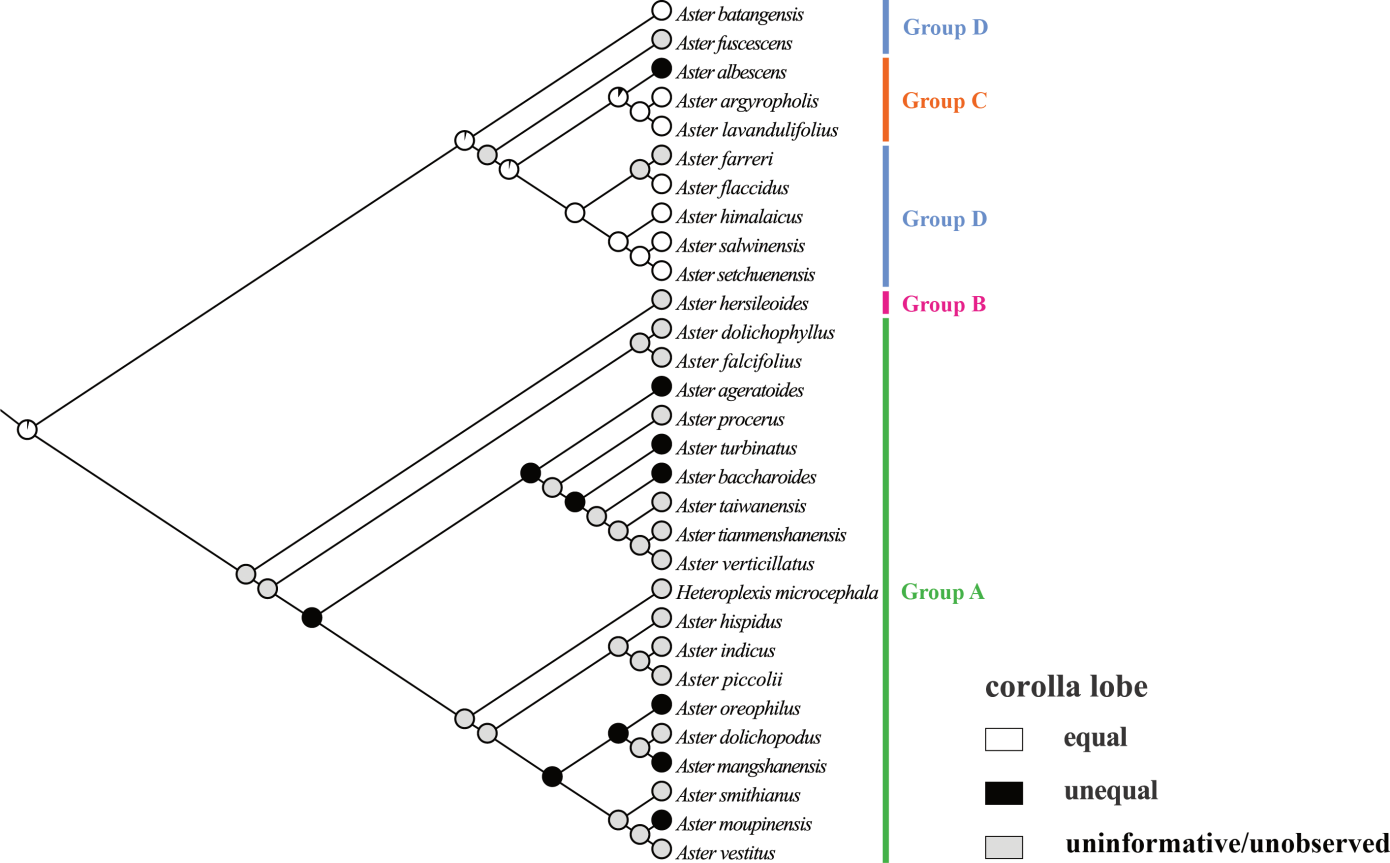
Ancestral state reconstruction of corolla lobe morphology using the method of likelihood ancestral states with the Mk1 model, optimized onto the phylogenetic tree inferred from internal transcribed spacer (ITS) sequences. “uninformative” indicates that no ancestral state inferred at this node due to missing trait data of descendant species. “unobserved” indicates that trait not observed for this species.

The equally lobed corolla may represent an ancestral trait (Fig. 7), although some uninformative nodes existed due to the unobserved trait in several species. This equally lobed type might also be the ancestral trait of groups C and D. Across the entire studied group, the equally lobed corolla (white circle) evolved independently three times (*A.lavandulifolius*, *A.flaccidus*, and *A.setchuenensis*). The unequally lobed corolla (black circle) evolved independently once (*A.mangshanensis*). From *A.batangensis* to *A.setchuenensis* (groups C and D), the unequally lobed corolla was only present in *A.albescens* (group C). This trait could be served as a species-level identification marker for *A.albescens*. However, the unequally lobed corolla quickly underwent a reversal, returning to the equally lobed form in *A.argyropholis*.

### ﻿Style base

**Figure 8.**
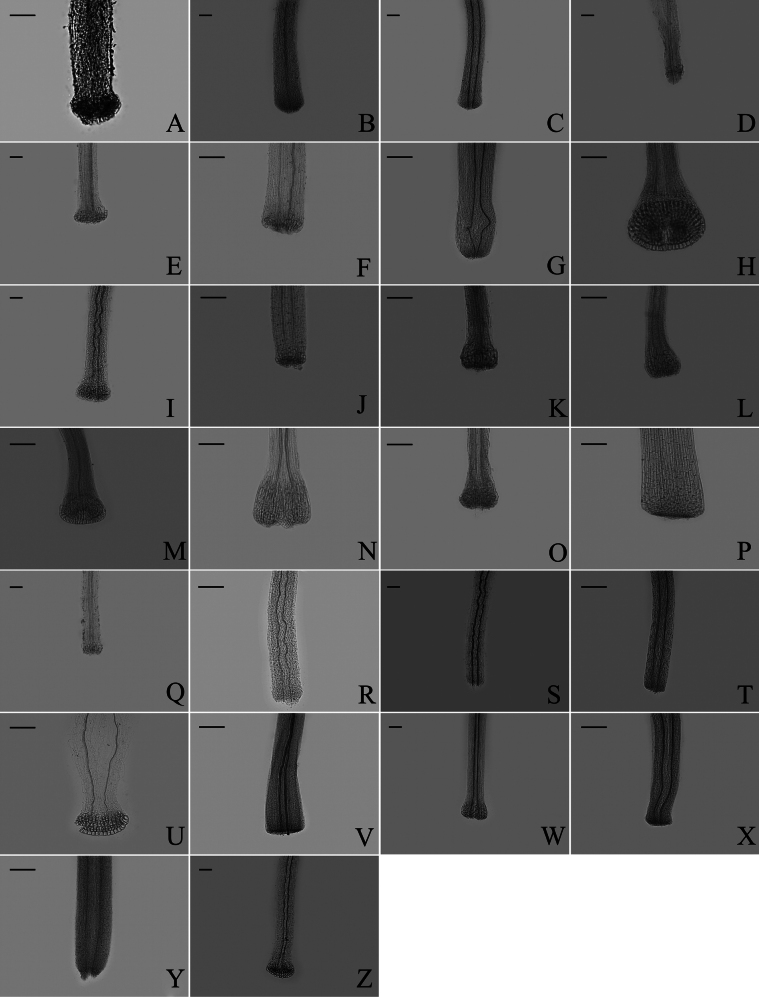
The morphology of style base in group A **A***A.smithianus***B***A.vestitus***C***A.altaicus***D***A.mangshanensis***E***A.oreophilus***F***A.ageratoides***G***A.verticillatus***H***A.turbinatus***I***A.piccolii***J***A.sinianus***K***A.giraldii***L***A.alatipes***M***A.moupinensis***N***A.falcifolius***O***A.taiwanensis***P***A.baccharoides***Q***A.brachytrichus***R***A.procerus***S***A.dolichopodus***T***A.tianmenshanensis***U***A.indicus***V***Heteroplexismicrocephala***W***A.tientschwanensis***X***A.sphaerotus***Y***A.dolichophyllus***Z***A.hispidus*. Scale bars: 200 μm.

**Figure 9.**
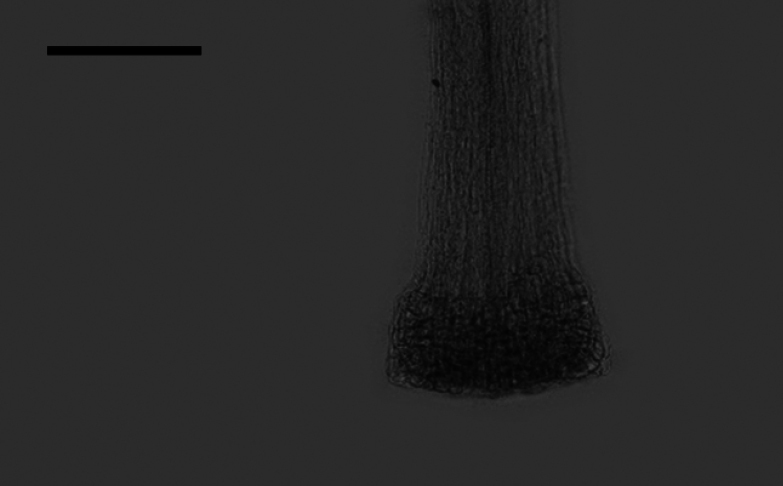
The morphology of style base in group B (*A.hersileoides*). Scale bar: 200 μm.

**Figure 10.**
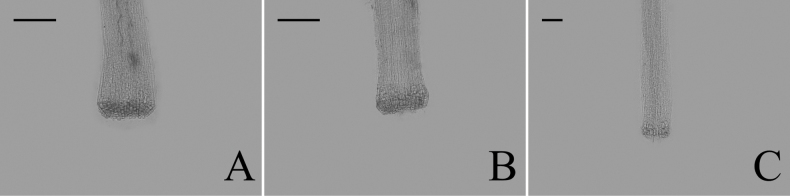
The morphology of style base in group C **A***A.lavandulifolius***B***A.albescens***C***A.hypoleucus*. Scale bars: 200 μm.

**Figure 11.**
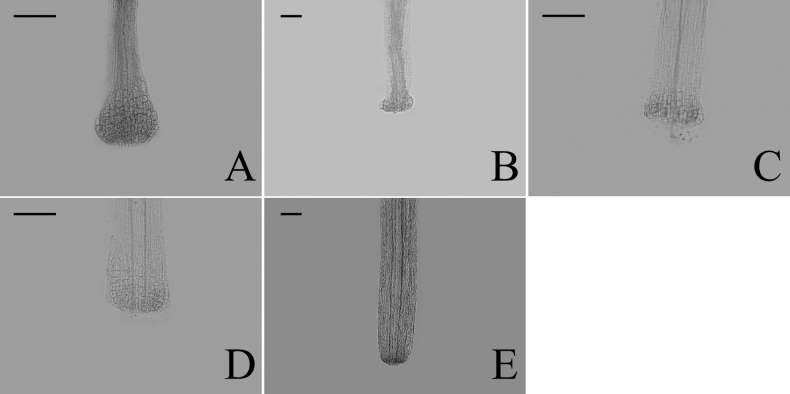
The morphology of style base in group D **A***A.batangensis***B***A.jeffreyanus***C***A.salwinensis***D***A.setchuenensis***E***A.prainii*. Scale bars: 200 μm.

Two types of style bases were identified in 40 sampled *Aster*. The first type was swollen, characterized by an abruptly swollen region of enlarged cells. The second type was unswollen, appearing as an enlarged area that either gradually tapers into the style shaft or maintains approx. the same diameter as the shaft. Within group A, the style base was swollen in 13 species (Fig. 8A, E, H, I, K–O, U, W, X, Z). The style base was unswollen in 13 species (Fig. 8B–D, F, G, J, P–T, V, Y). Within group B, the style base was swollen in *A.hersileoides* (Fig. 9). Within group C, the style base was unswollen in *A.lavandulifolius* (Fig. 10A), *A.albescens* (Fig. 10B), and *A.hypoleucus* (Fig. 10C). Within group D, the style base was swollen in *A.batangensis* (Fig. 11A) and *A.jeffreyanus* (Fig. 11B). The style base was unswollen in *A.salwinensis* (Fig. 11C), *A.setchuenensis* (Fig. 11D), and *A.prainii* (Fig. 11E).

**Figure 12.**
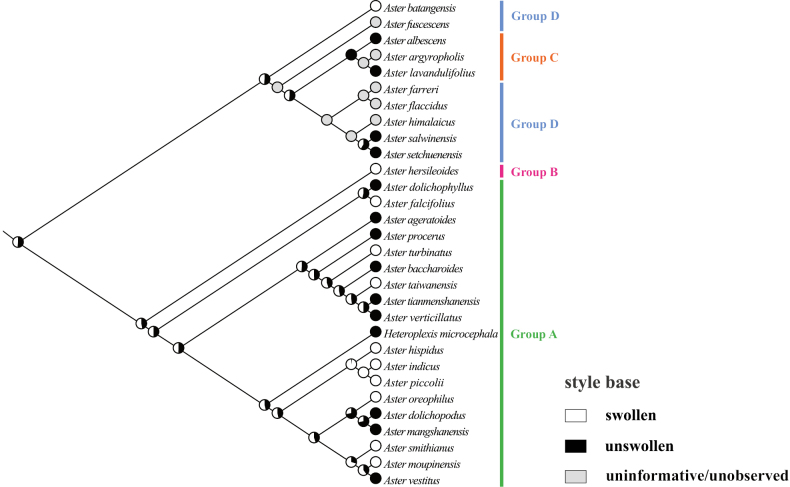
Ancestral state reconstruction of style base morphology using the method of likelihood ancestral states with the Mk1 model, optimized onto the phylogenetic tree inferred from internal transcribed spacer (ITS) sequences. “uninformative” indicates that no ancestral state inferred at this node due to missing trait data of descendant species. “unobserved” indicates that trait not observed for this species.

The ancestral trait of style base could not be identified (Fig. 12). The swollen style base (white circle) evolved independently twice (*A.falcifolius* and *A.piccolii*), with reversal to the unswollen type (black circle) occurring once (*A.ageratoides*). The unswollen style base evolved five times (*A.lavandulifolius*, *A.setchuenensis*, etc.), with two reversals to the swollen form (*A.hersileoides* and *A.smithianus*).

### ﻿Endothecium cell wall

**Figure 13.**
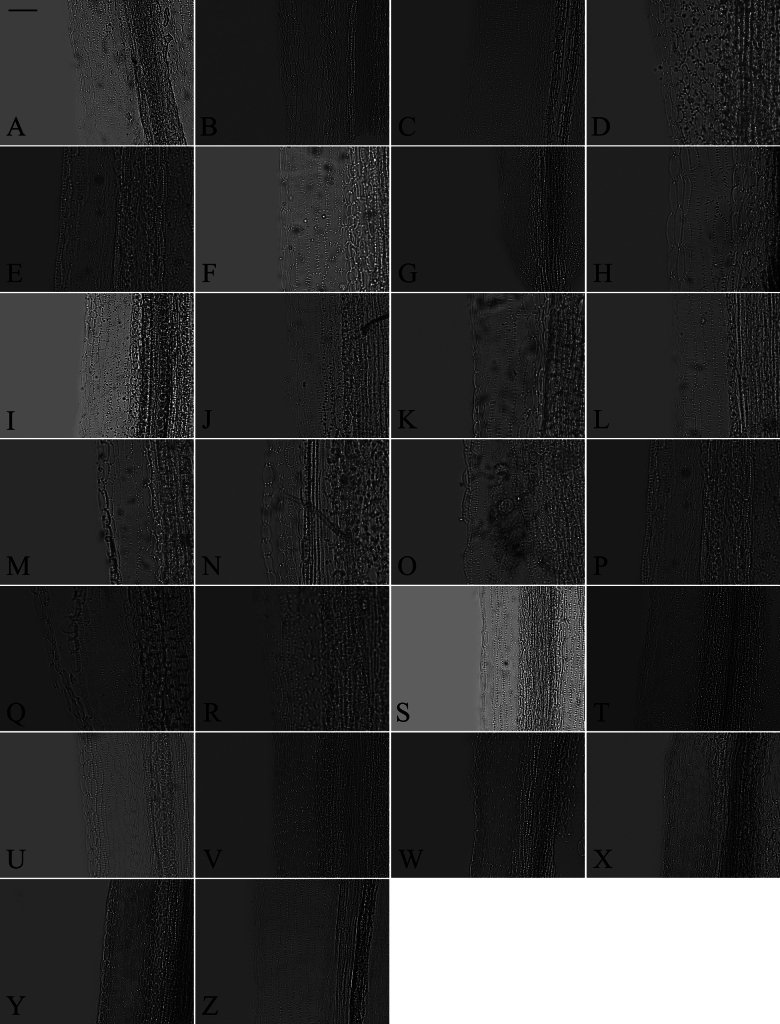
The morphology of anther endothecial cell wall thickenings in group A **A***A.smithianus***B***A.vestitus***C***A.altaicus***D***A.mangshanensis***E***A.oreophilus***F***A.ageratoides***G***A.verticillatus***H***A.turbinatus***I***A.piccolii***J***A.sinianus***K***A.giraldii***L***A.alatipes***M***A.moupinensis***N***A.falcifolius***O***A.taiwanensis***P***A.baccharoides***Q***A.brachytrichus***R***A.procerus***S***A.dolichopodus***T***A.tianmenshanensis***U***A.indicus***V***Heteroplexismicrocephala***W***A.tientschwanensis***X***A.sphaerotus***Y***A.dolichophyllus***Z***A.hispidus*. Scale bar: 50 μm.

**Figure 14.**
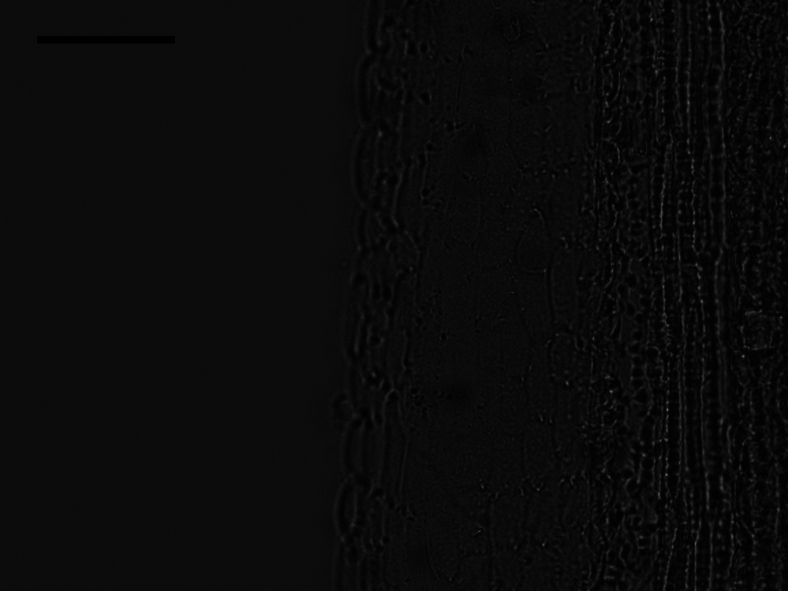
The morphology of anther endothecial cell wall thickenings in group B (*A.hersileoides*). Scale bar: 50 μm.

**Figure 15.**
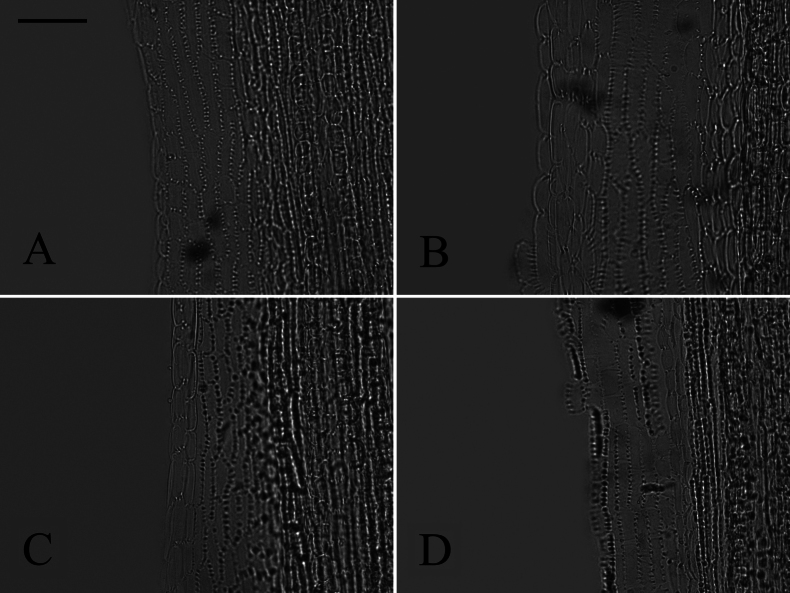
The morphology of anther endothecial cell wall thickenings in group C **A***A.lavandulifolius***B***A.albescens***C***A.hypoleucus***D***A.argyropholis*. Scale bar: 50 μm.

**Figure 16.**
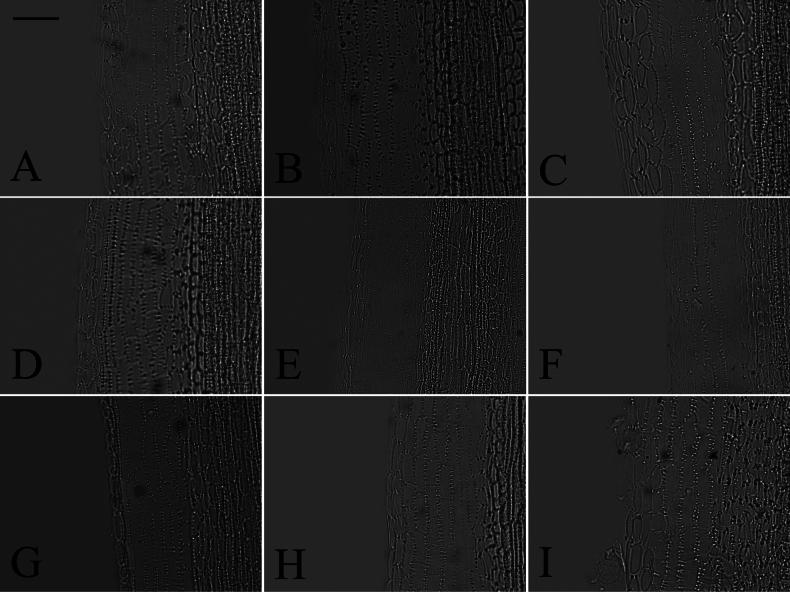
The morphology of anther endothecial cell wall thickenings in group D **A***A.batangensis***B***A.jeffreyanus***C***A.salwinensis***D***A.setchuenensis***E***A.prainii***F***A.fuscescens***G***A.farreri***H***A.flaccidus***I***A.himalaicus*. Scale bar: 50 μm.

Based on the different patterns of endothecium cell wall thickening, Dormer (1962) recognized three types: polarized (thickenings on either transverse or longitudinal walls), radial (cells ribbed all around), and transitional (uniformly thickening). In our study, the polarized and radial types of thickenings were present in group A (Fig. 13), group B (Fig. 14), group C (Fig. 15), and group D (Fig. 16). The transitional type was not observed. Within group A, *A.piccolii* (Fig. 13I), *A.taiwanensis* (Fig. 13O), and *A.dolichopodus* (Fig. 13S) had the radial type of thickening. The polarized type of thickening was present in 9 species (Fig. 13B, D, H, M, N, P, W, X, Y). Both polarized and radial types of thickenings were present in 14 species (Fig. 13A, C, E–G, J–L, Q, R, T, U, V, Z). Within group B, *A.hersileoides* (Fig. 14) had the polarized type of thickening. Within group C, *A.lavandulifolius* had the radial type of thickening (Fig. 15A), while both polarized and radial types of thickenings were present in *A.albescens* (Fig. 15B), *A.hypoleucus* (Fig. 15C), and *A.argyropholis* (Fig. 15D). Within group D, the polarized type of thickening was found in *A.salwinensis* (Fig. 16C), *A.farreri* (Fig. 16G), and *A.himalaicus* (Fig. 16I). Six species (Fig. 16A, B, D, E, F, H) showed both polarized and radial types of thickenings.

**Figure 17.**
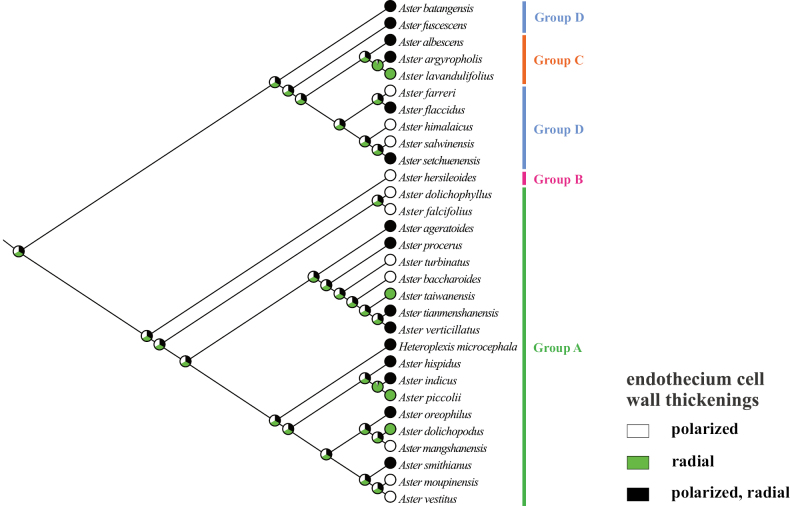
Ancestral state reconstruction of endothecium cell wall thickening morphology using the method of likelihood ancestral states with the Mk1 model, optimized onto the phylogenetic tree inferred from internal transcribed spacer (ITS) sequences.

The ancestral state for endothecium cell wall thickenings could not be inferred unequivocally in all groups (Fig. 17). The polarized type of thickening (white circle) evolved independently three times (*A.falcifolius*, *A.mangshanensis*, and *A.vestitus*), with two reversals (*A.ageratoides* and *A.smithianus*) to the combination type of polarized and radial (black circle). Within groups C and D, the radial thickening (green circle) was exclusively present in *A.lavandulifolius* and could similarly be used as a species-level identification character for this species. This trait evolved twice (*A.lavandulifolius*, and *A.piccolii*), with one reversal to the polarized type (*A.farreri*) and one reversal to the combination type (*A.oreophilus*). The combination type evolved three times (*A.flaccidus*, *A.setchuenensis*, and *A.verticillatus*) and underwent two reversals, returning to the polarized type (*A.himalaicus*, and *A.hersileoides*).

### ﻿Style branch

**Figure 18.**
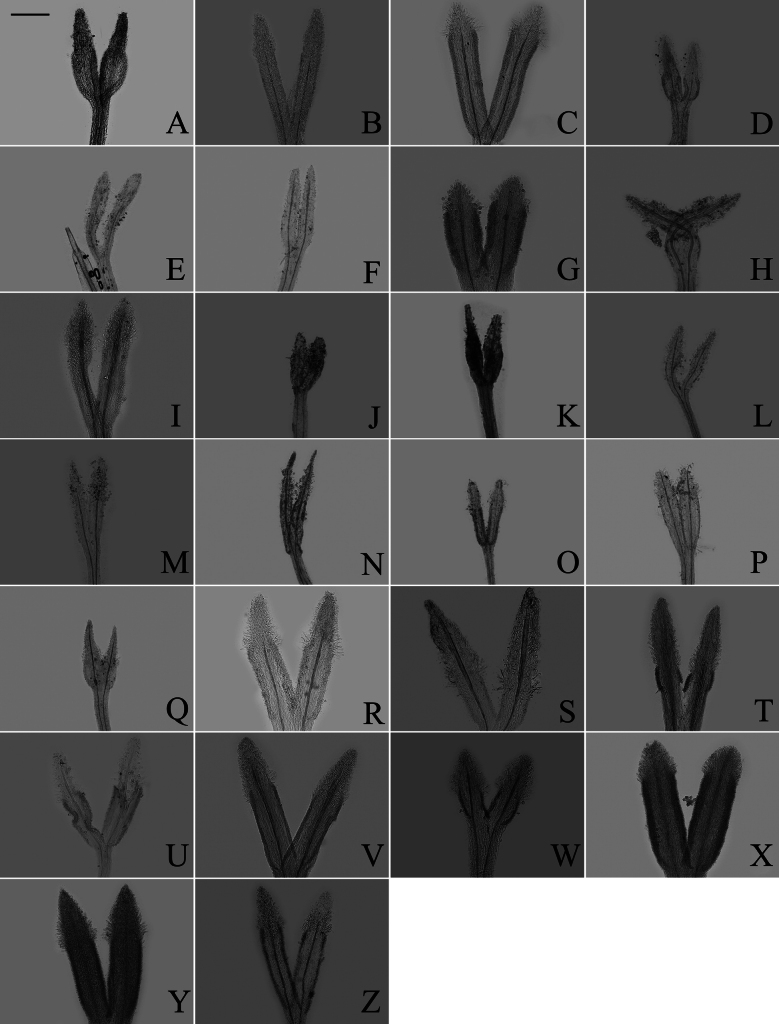
The morphology of style branches in group A **A***A.smithianus***B***A.vestitus***C***A.altaicus***D***A.mangshanensis***E***A.oreophilus***F***A.ageratoides***G***A.verticillatus***H***A.turbinatus***I***A.piccolii***J***A.sinianus***K***A.giraldii***L***A.alatipes***M***A.moupinensis***N***A.falcifolius***O***A.taiwanensis***P***A.baccharoides***Q***A.brachytrichus***R***A.procerus***S***A.dolichopodus***T***A.tianmenshanensis***U***A.indicus***V***Heteroplexismicrocephala***W***A.tientschwanensis***X***A.sphaerotus***Y***A.dolichophyllus***Z***A.hispidus*. Scale bar: 200 μm.

**Figure 19.**
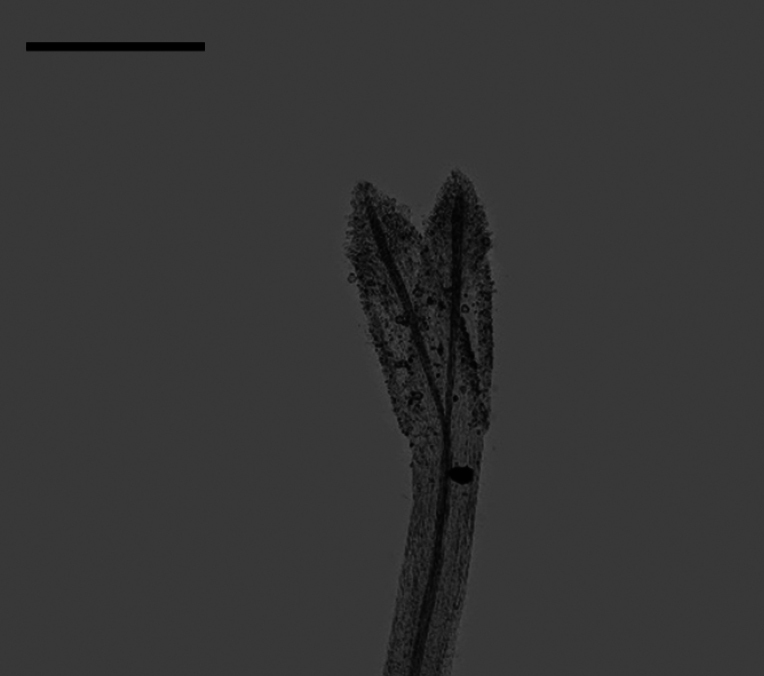
The morphology of style branches in group B (*A.hersileoides*). Scale bar: 200 μm.

**Figure 20.**
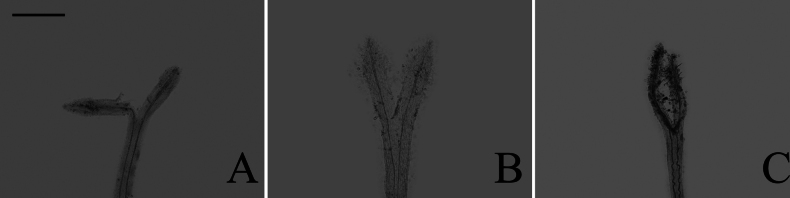
The morphology of style branches in group C **A***A.lavandulifolius***B***A.albescens***C***A.argyropholis*. Scale bar: 200 μm.

**Figure 21.**
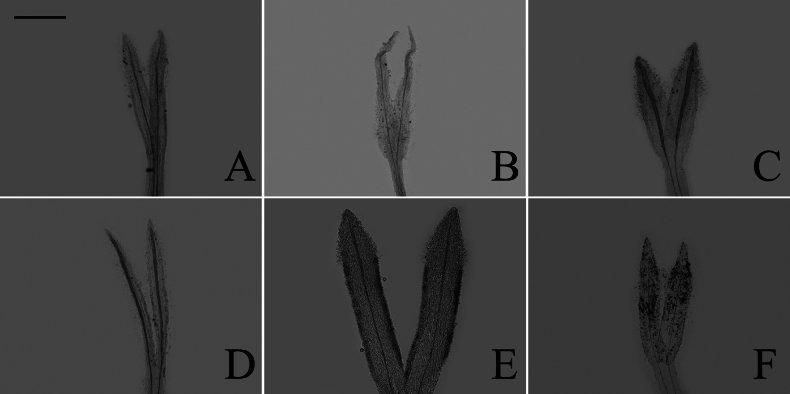
The morphology of style branches in group D **A***A.batangensis***B***A.jeffreyanus***C***A.salwinensis***D***A.setchuenensis***E***A.prainii***F***A.flaccidus*. Scale bar: 200 μm.

Three patterns of the relative length of the stigmatic line to the sterile tip appendage were observed: longer, shorter, and equal length. Within group A (Fig. 18), the length of stigmatic line was found to be longer than, shorter than, and equal to the length of the sterile tip appendage. Group B (Fig. 19) exhibited both the longer and equal length types, as group C (Fig. 20) only presented the equal length type. Within group D (Fig. 21), the longer, shorter, and equal length types were found. Within group A, the shorter length type was observed in *A.oreophilus* (Fig. 18E), *A.ageratoides* (Fig. 18F), *A.dolichopodus* (Fig. 18S), and *A.tianmenshanensis* (Fig. 18T). The equal length type was observed in 11 species (Fig. 18A, D, H, K–O, Q, U, W). The longer length type was observed in 11 species (Fig. 18B, C, G, I, J, P, R, V, X–Z). Within group B, *A.hersileoides* presented the longer length type (Fig. 19). Within group C, the longer length type was observed in *A.lavandulifolius* (Fig. 20A). The equal length type was observed in *A.albescens* (Fig. 20B) and *A.argyropholis* (Fig. 20C). Within group D, the shorter length type was observed in *A.jeffreyanus* (Fig. 21B). The equal length type was observed in *A.salwinensis* (Fig. 21C) and *A.setchuenensis* (Fig. 21D). The longer length type was observed in *A.batangensis* (Fig. 21A) and *A.prainii* (Fig. 21E).

**Figure 22.**
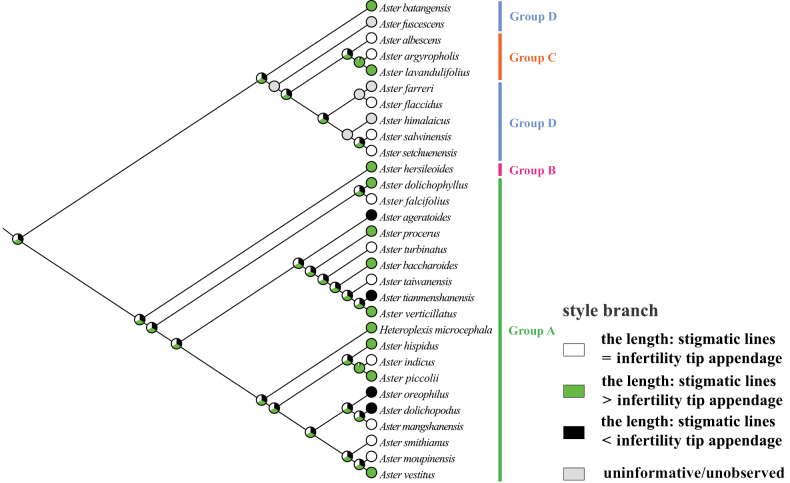
Ancestral state reconstruction of style branch morphology using the method of likelihood ancestral states with the Mk1 model, optimized onto the phylogenetic tree inferred from internal transcribed spacer (ITS) sequences. “uninformative” indicates that no ancestral state inferred at this node due to missing trait data of descendant species. “unobserved” indicates that trait not observed for this species.

The ancestral state for style branch could not be unambiguously identified across all groups (Fig. 22). The equal length type (white circle) evolved independently four times (*A.flaccidus*, *A.setchuenensis*, etc.) and quickly returned three times to the longer type (*A.hersileoides*) or the shorter type (*A.ageratoides*, and *A.oreophilus*). The longer length type (green circle) evolved four times (*A.lavandulifolius*, *A.verticillatus*, etc.), with one reversal to the shorter type (*A.dolichopodus*). Notably, *A.lavandulifolius* exhibited a uniquely longer relative length in group C (mainly equal length type). This state could likewise be employed as a species-level identification trait for this species. The shorter length type (black circle) evolved independently once (*A.oreophilus*) and quickly reverted, returning to the equal type (*A.smithianus*).

## ﻿Discussion

### ﻿Phylogenetic analysis

Due to morphological similarity among distantly related groups, the extent of *Aster* has been the subject of great controversy and a well-known taxonomic conundrum. Currently, Chen et al. (2024) divided *Aster* into clades A–D, corresponding to groups A–D recognized in this study (Table 1). Their results also suggested the inclusion of the genera *Kalimeris*, *Heteropappus*, *Heteroplexis* and others within *Aster*. Nesom (2020a, 2020b, 2020d, 2020f, 2020h, 2020i) segregated multiple new combinations (Table 1) from *Aster*, namely *Chlamydites*, *Geothamnus*, *Griersonia*, *Sinosidus*, *Tibetiodes*, *Yonglingia*, etc. Chen et al. (2024) suggested that groups B–D might warrant recognition as independent genera, thereby supporting the views of Nesom (2020b, 2020f, 2020h, 2020i) that several clades within *Aster* should be elevated to the generic level. Our Phylogenetic tree indicated that core *Aster* is a monophyletic group, aligning with the findings of Li et al. (2012) and Chen et al. (2024). It also supported the placement of *Gymnaster* (Ito et al. 1995; Chen et al. 2011; Li et al. 2012; Nesom 2020d, 2020g), *Heteropappus* (Ito et al. 1998; Li et al. 2012; Nesom 2020d, 2020g; Duan et al. 2022; Chen et al. 2024), *Heteroplexis* (Nesom 2020d; Duan et al. 2022; Chen et al. 2024), *Kalimeris* (Ito et al. 1995; Ito et al. 1998; Chen et al. 2011; Li et al. 2012; Nesom 2020d, 2020g; Chen et al. 2024), and *Rhynchospermum* (Bremer and Anderberg 1994; Chen et al. 2011; Nesom 2020d, 2020g) within core *Aster*. However, three new combinations previously segregated from core *Aster* by Nesom conflicted with our phylogenetic results, namely *Cardiagyris* (Nesom 2020a), *Chaochienchangia* (Nesom 2020i), and *Tibetiodes* (Nesom 2020h). For instance, *Tibetiodes* comprises both Alpine *Aster* species (group D) and several taxa nested within core *Aster* (group A). Therefore, we currently do not support Nesom’s subdivision of core *Aster*, as this would introduce unnecessary nomenclatural complexity and taxonomic instability. Broader sampling and additional evidence will be required in future studies to determine whether core *Aster* warrants subdivision into multiple smaller genera.

In our phylogenetic tree (Fig. 3), Asterser.Hersileoides (*A.hersileoides*) formed a distinct lineage on its own and exhibited a sister relationship with core *Aster* with low support (PP = 0.55). This could be interpreted that Asterser.Hersileoides might should be recognized as an independent genus in Astereae, in agreement with the studies of Chen et al. (2011), Li et al. (2012), and Nesom (2020d, 2020i). In the *Flora of China*, Asterser.hersileoides was treated as an unplaced *Aster* group (Chen et al. 2011). Li et al. (2012) found that Asterser.hersileoides formed a well-supported monophyletic group. Nesom (2020i) treated this series as a new genus *Yonglingia* in Astereae. Within Alpine *Aster*, species were clustered into three lineages, potentially supporting the segregation of these clades into three new genera. Within our sampled taxa, Nesom (2020h) proposed the reclassification of *A.salwinensis*, *A.setchuenensis*, *A.himalaicus*, *A.farreri*, and *A.flaccidus*, placing them into *Tibetiodes*. Similarly, Nesom (2020f) segregated *A.fuscescens* into *Griersonia*. *A.batangensis* formed a separate lineage as the basal group of *Aster*, distinct from other taxa of Alpine *Aster*. This finding aligned with Li et al. (2012), Nesom (2020h), and Chen et al. (2024). It further supported the possible segregation of *A.batangensis* into a new genus *Geothamnus* proposed by Nesom (2020h). Compared to core *Aster*, Asterser.Albescentes and Alpine *Aster* displayed a closer phylogenetic relationship, collectively forming a clade (except for *A.batangensis*), consistent with the finding of Li et al. (2012). Although nested within Alpine *Aster*, Asterser.Albescentes formed a monophyletic group, aligning with previous studies (Li et al. 2012; Nesom 2020f). Chen et al. (2011) recognized this series as an unplaced *Aster* group. Li et al. (2012) proposed elevating Astersect.Alpigenia to the genus level based on molecular evidence, and suggested that Asterser.Albescentes was more closely related to Astersect.Alpigenia. Nesom (2020d, 2020f) segregated this series from *Aster* and treated it as a new combination namely *Sinosidus*, based on its morphology traits and phylogenetic position. Overall, the results supported a distant relationship among Asterser.Hersileoides, Asterser.Albescentes, Alpine *Aster* (including *A.batangensis*), and core *Aster*. This also suggested that the first three groups (groups B–D) should be considered as separate genera. However, given the limited taxa sampling in this study, further morphology and molecular evidence are required to support the segregation of groups B–D into multiple genera in Astereae.

### ﻿The analysis of floral micromorphology

#### ﻿Corolla lobe

Both equally and unequally lobed corolla were present in groups A and C, with no regular variation observed (Table 1, Fig. 7). However, group D presented only equally lobed corolla, showing a high degree of consistency. The similarity may be considered a synapomorphy. This character might be useful for diagnosing group D and delineate it from other taxa in *Aster*. Li et al. (2012) supported Alpine *Aster* did not form a monophyletic lineage with the other groups of *Aster* using ITS, ETS, and *trnL-F* sequences. Nesom (2020b, 2020f, 2020h) also supported the independence of Alpine *Aster* from *Aster* and proposed several new genera, such as *Chlamydites*, *Geothamnus*, and *Tibetiodes*. Our morphological result was consistent with the phylogenetic analysis, also supporting that the Alpine *Aster* (group D) should be kept separately from *Aster*. However, large data gaps remain within group D. Therefore, further sampling of group D is needed in future research.

#### ﻿Style base

The style base has been widely used to delimit subtribes and genera in the tribe Eupatorieae (Robinson and King 1977) and Senecionea (Robinson and Brettell 1973). Swollen and unswollen types of style base were all found in groups A and D (Table 1, Fig. 12). Obviously, the trait of style base does not support the definition of groups A and D. Wetter (1983) concluded that the style base was highly variable, aligned with our study. Scott (1985) similarly argued that the style base was taxonomically useless in Astereae. However, we found that group C (Asterser.Albescentes) presented only unswollen style base, showing a high degree of consistency. This trait may be appropriate to delineate group C from other taxa in *Aster*. Nesom (1994a) suggested that group C might not belong to *Aster* based on its habit, involucres, and achenes. Molecular systematics studies of Li et al. (2012) and Chen et al. (2024) found that group C clustered into a highly supported monophyletic taxon. These studies also concluded that group C should be removed from *Aster* and considered as a separate genus. Nesom (2020f) described a new genus, *Sinosidus* Nesom gen. nov., based on previous morphological studies (Nesom 1994a, 2020d, 2020g) and molecular phylogenetic studies (Li et al. 2012; Zhang et al. 2015, 2019; Fu et al. 2019). Our phylogenetic and morphological results also supported the independence of group C. However, as large data gaps persist within group D, further sampling efforts are necessary in future studies.

### ﻿Endothecium cell wall

The endothecium cell is oblong or square in the family Asteraceae. Liu and Yang (2010) demonstrated that the types of cell wall thickening exhibit distinct regularity among genera, providing valuable characters for classifying subtribes and genera. However, in our study, both polarized and radial types of thickening were present in all groups, making infrageneric differentiation in *Aster* unreliable (Table 1, Fig. 17). Similarly, Robinson and Brettell (1973) found that anther exothecial cell wall thickening of *Senecio* (Senecioneae) and its close relatives was not very reliable. Karis (1993) reported that the polarized type of thickening occurs extensively across the most genera of Astereae. However, in our study, the different types of thickening in *Aster* did not support the study of Karis (1993). Therefore, this trait is not reliable for classification of *Aster*.

### ﻿Style branch

Bremer and Anderberg (1994) identified the style branch morphology as an important trait for the classification of Astereae. The style branch was highly consistent within Astereae, typically being triangular lanceolate with sterile appendages at the tip. Our observations revealed that the length of the stigmatic line relative to the sterile appendage varies significantly among groups (Table 1, Fig. 22). Therefore, it is not a reliable character for the delimiting *Aster*.

### ﻿Reconstruction of ancestral traits

Asteraceae species exhibits complex group relationships and diverse morphological variation. Understanding the species variation and developmental patterns is crucial for exploring morphological evolution, key innovative traits, and evolutionary potential within the family (Pozner et al. 2012). In this study, ancestral trait reconstruction of four traits of floral micromorphology was conducted for *Aster* to facilitate a more intuitive observation of the evolutionary patterns of these traits. The results of ancestral state reconstruction revealed that many traits evolved independently multiple times and underwent several reversals, returning to different types. This may contribute to their complexity and irregular variation. Coupled with the significant data gaps (unobserved traits) for corolla lobe and style base, these factors posed challenges for reconstruction. Although equally lobed corolla was likely an ancestral trait (Fig. 7), the presence of many uninformative nodes decreased the reliability of the result. Moreover, the ancestral traits of style base (Fig. 10), endothecium cell wall thickenings (Fig. 15), and style branch (Fig. 20) could not be reconstructed successfully. To improve ancestral state reconstruction, future work should aim to broaden taxon sampling and address data gaps in floral micromorphological traits. However, the inability to effectively reconstruct ancestral traits suggested that the systematic significance of floral micromorphology within core *Aster* and its related groups is restricted.

This study compared floral microcharacters and previous taxonomic treatments of *Aster* (Nesom 1994a, 1994b; Chen et al. 2011; Li et al. 2012; Nesom 2020d, 2020f; Chen et al. 2024) (Table 2). These four floral microcharacters did not correspond to the traditionally recognized boundaries of *Aster*. Based on four floral microcharacters alone, *Aster* cannot be strictly classified into certain taxa. Similarly, a single floral microcharacter does not evidently support a division of groups. Using floral micromorphology to delimit genera would require a major restructuring of *Aster*, possibly obscuring species relationships. Although these traits are not yet suitable for infrageneric classification, they appear to be feasible and reliable for species- or group-level identification within core *Aster* and its related groups. For instance, *A.mangshanensis* was well distinguished from other taxa of group A by the combination of features such as unequally lobed corolla, unswollen style base, polarized type of thickening, and the equal length of the stigmatic line to the apical sterile appendage of style branch. Similarly, in group C, the features of unequally lobed corolla, unswollen style base, both polarized and radial types of thickening, and the equally relative length of the stigmatic line to the sterile appendage of style branch could differentiate *A.albescens* from other species. *A.salwinensis* exhibited equally lobed corolla, unswollen style base, polarized type of thickening, and the equally relative length of the stigmatic line to the sterile appendage. This combination of characters made *A.salwinensis* well distinguishable from other taxa of group D. Other useful traits include radial thickening and longer relative length in *A.lavandulifolius*, unswollen style base in group C, and equally lobed corolla in group D.

In the sampled core *Aster* herein, the type species of the formerly genera *Heteropappus* (*A.hispidus*), *Kalimeris* (*A.indicus*), and *Rhynchospermum* (*A.verticillatus*) were included (Table 1). Due to sampling difficulties and time constraint, the type species of the genera *Aster* (*Asteramellus* L.), *Gymnaster* (*Gymnastersavatieri* (Makino) Kitam. = *Astersavatieri* Makino) and *Heteroplexis* (*Heteroplexisvernonioides* C. C. Chang) were not included. These genera (except *Heteroplexis*) had been formally revised and synonymized as *Aster*. Regarding *A.amellus*, Nesom (2020d) included it within the *Asteramellus* group at the terminal branch of phylogenetic clade, including *A.alpinus*, *Rhynchospermum*, *Turczaninovia*, and several species with unresolved placement. Regarding *Gymnaster*, it was originally the name of the genus later recognized as *Miyamayomena*. Ito et al. (1995) treated *M.savatieri* (type species of *Gymnaster*) as a species of Astersect.Teretiachaenium. However, Ito et al. (1998) and Li et al. (2012) found the polyphyly of *Miyamayomena*. Li et al. (2012) proposed the subsumption of three Chinese *Miyamayomena* species into *Aster*, keeping *M.savatieri* and *Miyamayomenakoraiensis* (Nakai) Kitam. (type speceis of *Miyamayomena*) unresolved. In contrast, Nesom (2020d) placed *Miyamayomena* in the *Kalimeris* group within *Aster*. Regarding *Heteroplexis*, phylogenetic studies of Nesom (2020d), Duan et al. (2022), and Chen et al. (2024) consistently concluded its inclusion within *Aster*. Notaby, Duan et al. (2022) proposed the subsumption of *Heteroplexis* into *Aster* based on phylogenetic evidence of the nesting of *H.vernonioides* and its 4 relatives within *Aster*. Therefore, based on previous studies, the unsampled type species are most likely positioned within core *Aster*. Moreover, previous studies have made significant contributions to classical taxonomy and molecular systematics of *Aster* (Ling et al. 1985; Nesom 1994a, 1994b; Chen et al. 2011; Li et al. 2012; Jafari et al. 2015; Korolyuk et al. 2015; Zhang et al. 2015, 2019; Nesom 2020a, 2020b, 2020c, 2020d, 2020e, 2020f, 2020g, 2020h, 2020i; Chen et al. 2024). These studies concluded that *Aster* currently defined is not a monophyletic taxon and requires reclassification. This added more phylogenetic issues to be solved, such as the subdivision of *Aster* and the taxonomic treatments of Alpine *Aster*, Asterser.Hersileoides, and Asterser.Albescentes. In the future, the integration of morphology, anatomy, and molecular systematics will be crucial to addressing the taxonomic challenges within *Aster*. Additionally, *Aster* currently defined is estimated to include 152–180 species, yet this study examined only 40 of them. Therefore, a thorough sampling of floral micromorphological characters, combined with other data types, is essential for a comprehensive and formal revision of core *Aster* and its related groups.

## ﻿Additional information

### Author ORCIDs

Xinyu Chen https://orcid.org/0009-0008-6347-2490

Tianmeng Qu https://orcid.org/0009-0008-0185-5306

Xinyi Zheng https://orcid.org/0009-0003-2300-5525

Yanru Zhang https://orcid.org/0009-0009-0450-2001

Hui Chen https://orcid.org/0009-0003-4786-7453

Bo Li https://orcid.org/0000-0002-5131-8639

Liang Dou https://orcid.org/0009-0009-8200-5721

Zhixi Fu https://orcid.org/0000-0002-2789-6287

### Data availability

The dataset of ITS sequences analyzed herein is available in the NCBI GenBank database. The accession numbers of sequences used are provided in Table 3 of the main text. The data of floral micromorphology generated in this study are also provided in Tables 2, 4 and figures of the main text.
